# Selective Surface and Intraluminal Localization of Wnt Ligands on Small Extracellular Vesicles Released by HT-22 Hippocampal Neurons

**DOI:** 10.3389/fcell.2021.735888

**Published:** 2021-10-13

**Authors:** Viviana I. Torres, Daniela P. Barrera, Manuel Varas-Godoy, Duxan Arancibia, Nibaldo C. Inestrosa

**Affiliations:** ^1^Departamento Bioquímica y Biología Molecular, Facultad de Ciencias Biológicas, Universidad de Concepción, Concepción, Chile; ^2^Centro de Envejecimiento y Regeneración (CARE UC), Departamento de Biología Celular y Molecular, Facultad de Ciencias Biológicas, Pontificia Universidad Católica de Chile, Santiago, Chile; ^3^Cancer Cell Biology Laboratory, Centro de Biología Celular y Biomedicina (CEBICEM), Facultad de Medicina y Ciencia, Universidad San Sebastián, Santiago, Chile; ^4^Departamento Biomédico, Facultad de Ciencias de la Salud, Universidad de Antofagasta, Antofagasta, Chile; ^5^Centro de Excelencia en Biomedicina de Magallanes (CEBIMA), Universidad de Magallanes, Punta Arenas, Chile

**Keywords:** exosomes, hippocampal cells, porcupine, Wnt-C59, Wnt ligand

## Abstract

The Wnt signaling pathway induces various responses underlying the development and maturation of the nervous system. Wnt ligands are highly hydrophobic proteins that limit their diffusion through an aqueous extracellular medium to a target cell. Nevertheless, their attachment to small extracellular vesicles-like exosomes is one of the described mechanisms that allow their transport under this condition. Some Wnt ligands in these vehicles are expected to be dependent on post-translational modifications such as acylation. The mechanisms determining Wnt loading in exosomes and delivery to the target cells are largely unknown. Here, we took advantage of a cell model that secret a highly enriched population of small extracellular vesicles (sEVs), hippocampal HT-22 neurons. First, to establish the cell model, we characterized the morphological and biochemical properties of an enriched fraction of sEVs obtained from hippocampal HT-22 neurons that express NCAM-L1, a specific exosomal neuronal marker. Transmission electron microscopy showed a highly enriched fraction of exosome-like vesicles. Next, the exosomal presence of Wnt3a, Wnt5a, and Wnt7a was confirmed by western blot analysis and electron microscopy combined with immunogold. Also, we studied whether palmitoylation is a necessary post-translational modification for the transport Wnt in these vesicles. We found that proteinase-K treatment of exosomes selectively decreased their Wnt5a and Wnt7a content, suggesting that their expression is delimited to the exterior membrane surface. In contrast, Wnt3a remained attached, suggesting that it is localized within the exosome lumen. On the other hand, Wnt-C59, a specific inhibitor of porcupine O-acyltransferase (PORCN), decreased the association of Wnt with exosomes, suggesting that Wnt ligand acylation is necessary for them to be secreted by exosomes. These findings may help to understand the action of the Wnt ligands in the target cell, which could be defined during the packaging of the ligands in the secretory cell sEVs.

## Introduction

Since its discovery, the Wnt signaling and related molecules have been shown to be essential for controlling the development and maturation of the central nervous system (CNS). Wnt ligands modulate several processes during development, such as neurogenesis, synapse formation, synaptic plasticity, and neuronal survival (Dickins and Salinas, 2013; Inestrosa and Varela-Nallar, 2015; McLeod and Salinas, 2018; Oliva et al., 2018). At an early developmental stage, failure of Wnt signaling can compromise synapse formation and consequently the establishment of neuronal circuits (Noelanders and Vleminckx, 2017; Torres et al., 2017; Oliva et al., 2018). In the classical mechanism of action Wnt ligands bind to extracellular receptors activating at least two pathways: the canonical or Wnt/β-catenin pathway and the non-canonical or independent β-catenin pathway (Niehrs, 2012; Nusse and Clevers, 2017). In the canonical β-catenin dependent pathway, Wnt proteins exert their function by binding to the extracellular domains of Frizzled receptors (Fz)/LRP5/6 co-receptor (MacDonald and He, 2012) and in the β-catenin independent pathway, Wnts bind RYK/Derailed, and ROR receptors (Green et al., 2014).

After synthesis, Wnt molecules undergo glycosylation and acylation at the endoplasmic reticulum (ER) and follow the secretory pathway (Takada et al., 2006; Herr and Basler, 2012; Torres et al., 2019). The role of glycosylation in secretion appears to be specific to the Wnt ligand. For example, it has been reported in a mouse cell line that any of the four asparagine(N)-linked glycosylations of Wnt1 are necessary for secretion (Mason et al., 1992). A similar observation was made in *Drosophila* with the Wnt homolog Wingless (Wg; Tang et al., 2012). On the contrary, site-directed mutagenesis of the four asparagine residues in Wnt5a did affect secretion into the extracellular matrix (ECM; Kurayoshi et al., 2007; Torres et al., 2019). The other modification that Wnt molecules undergo is the addition of a lipid moiety by the O-acyltransferase PORCN, which adds palmitoleic acid to specific serine residues of Wnts (Gao and Hannoush, 2014; Torres et al., 2019). Although several studies have suggested that acylation mediated by PORCN is necessary for Wnt secretion (Kadowaki et al., 1996; Barrott et al., 2011; Biechele et al., 2011), the latest findings in several cancer cells lines and a specific T cell seem to contradict that (Richards et al., 2014; Rao et al., 2019). A putative explanation for those opposed results is that Wnt acylation mediated by PORCN is only necessary for their association with specific Wnt transporters.

Once Wnts reach the extracellular milieu, they can act in an autocrine as well as in a paracrine fashion. In the latter, Wnt ligands act over long distances from a Wnt-producing cell (Zecca et al., 1996; Neumann and Cohen, 1997). Given this capability, it was hypothesized that instead of spreading to reach their targets, they could be transferred between cells by different mechanisms, which include: (i) specialized filopodia mechanism called cytonemes (Takada et al., 2017); (ii) lipoproteins or the SWIM protein (Mulligan et al., 2012; Kaiser et al., 2019); (iii) membranous structures designated as argosomes (Greco et al., 2001) which are lipoprotein particles surrounded by lipid particles; and (iv) small extracellular vesicles (sEVs) referred to as exosomes (Gross et al., 2012; Działo et al., 2019). These extracellular vesicles have a diameter of 50–150 nm, are secreted by most cell types (Raposo and Stoorvogel, 2013), and deliver macromolecules to their target cells in healthy and pathological conditions (Isola and Chen, 2017). What determines the extracellular transport mechanisms for Wnts is unclear which might be tissue, cell type or Wnt specific, but also the physiological stage of the secreting cell might play a role.

In the CNS, microglia, oligodendrocyte and neurons release exosomes (Potolicchio et al., 2005; Fauré et al., 2006; Krämer-Albers et al., 2007) and *in vitro* evidence demonstrates that changes in synaptic activity modulate the release of exosomes from neurons (Fauré et al., 2006; Lachenal et al., 2011). These exosomes can be reincorporated into other neurons, suggesting a new form of inter-neuronal communication. In *Drosophila*, Wg is associated with exosomes, and the protein GPR177/Evi transfers it from the presynapse to the postsynapse (Korkut et al., 2009; Beckett et al., 2013). In vertebrates, Wnt ligands also co-localize with exosomes (Gross et al., 2012; Działo et al., 2019) in physiological and pathological conditions (Zhang and Wrana, 2014; Działo et al., 2019), but in the CNS, to the best of our knowledge, there are no reports on neurons releasing Wnt containing exosomes.

Although Wnt molecules were described 40 years ago, many questions remain unanswered regarding the mechanism of intracellular sorting into the different putative cargoes, and less is known about the possible role of exosomes in mediating Wnt signaling protein transport from one cell to another. Here, we determined the association between Wnt ligands and sEVs. Also, we described how the inhibition of Wnt ligands palmitoylation affects their release associated with these vesicles. Finally, we show the topological localization of Wnt signaling proteins in the population of sEVs released from HT-22 cells.

## Materials and Methods

This study was not pre-registered. No blinding was performed. Institutional Ethical Approval was not required for the study.

### Cell Culture and Small Extracellular Vesicle Preparation

The immortalized mouse hippocampal cell line HT-22 (passages 15–20) was cultured with the following Thermo Fisher Scientific Inc., reagents: Dulbecco’s Modified Eagle Medium (Catalog Number: 11966025) supplemented with 4.5 g/L glucose, 10% Fetal Bovine Serum (FBS) (Catalog Number: 16000044), 2 mM glutamine (Catalog Number: 25030024), 100 I.U./ml penicillin-100 μg/ml Streptomycin (Catalog Number: 15140163), at 37°C under 5% CO_2_. Once 60–70% confluency was reached, cells were washed twice with phosphate buffer saline (PBS) to remove any FBS. Then, cells were grown in the same medium but using 10% exosome depleted FBS (Thermo Fisher Scientific Inc., Catalog Number: A2720803). After 48 h, the conditioned medium (CM) of HT-22 cells was collected, and the cells were lysed with RIPA buffer containing Halt protease inhibitor cocktail (Thermo Fisher Scientific Inc., Catalog Number: 78445). The CM was subjected to three consecutive centrifugations: 2,000× *g*, 10 min; 20,000× *g*, 20 min; and 100,000× *g*, for 2 h at 4°C. The final pellet, called P100 or EV (Kowal et al., 2016), was resuspended in 0.2 μm filtered PBS, pelleted again, and stored at −80°C until further analysis or used immediately for downstream analysis.

### Electron Microscopy

P100 was re-suspended in PBS, re-pelleted and analyzed by transmission electron microscopy. To examine its fine structure, P100 was fixed in 2.5% glutaraldehyde/0.1 M sodium cacodylate buffer pH 7.4 at room temperature overnight (EMS, Catalog Number: 15960). Then it was washed with cacodylate buffer for 2 h and post-fixed with 1% aqueous osmium tetroxide for 2 h, rinsed with bidistilled water and stained with 1% uranyl acetate for 90 min. It was dehydrated twice stepwise with increasing acetone concentrations (50, 70, 95, and 100%) for 30 min each. Preinclusion was done with epon:acetone (1:1) overnight and then it was included in pure epon. The polymerization was carried out in an oven at 60°C for 48 h. Fine cuts of 80 nm thickness were obtained in a Leica Ultracut R Ultramicrotome, stained with 4% uranyl acetate in methanol for 2 min and with Reynolds’s lead citrate for 5 min. The slices were observed in a Philips Tecnai 12 BioTwin microscope (Eindhoven, Netherlands) at 80 kV. All post-fixation treatments of a sample were performed in the advanced Pontificia Universidad Católica de Chile Microscopy Unit.

### Immunogold

For immunogold analysis, P100 pellets were fixed in 4% paraformaldehyde (PFA) in 0.1 M phosphate buffer pH 7.0, 0.2% for at 4°C for 2 h, then rinsed for 30 min in phosphate buffer, dehydrated with 50, 70, 95, and 100% ethanol for 15 min each and then left overnight in ethanol/LR White 1:1. Then, P100 pellets were included in the resin in gelatin capsules and polymerized at 50°C for 8 h. A Leica Ultracut R Ultramicrotome was used to make fine 90 nm wide cuts, which were placed in nickel grids. A cut was blocked with PBS/0.1% BSA for 30 min, then incubated with a 1:20 dilution of a primary antibody (CD63, Santa Cruz Biotechnology, Inc., Catalog Number: sc-5275; Wnt3a, Thermo Fisher Scientific Inc., Catalog Number: PA5-44946; Wnt5a, Abcam, Catalog Number: ab174963; Santa Cruz Biotechnology, Inc., Wnt5a Catalog Number: sc-365370; Thermo Fisher Scientific Inc., Wnt7a, Catalog Number: PA5-80231; MyBioSource GPR177, Catalog Number: MBS769833, and washed three times with PBS/0.1% BSA/0.2% Triton X-100 for 20 min. Goat anti-Rabbit Gold 6 nm, (Abcam, Catalog Number: ab41498) or goat anti-mouse Gold 25 nm (EMS, Catalog Number: 25135) was used as secondary antibody and incubated for 1 h, then washed three times for 20 min with PBS/0.1% BSA/0.2% Triton X-100, and a rapid final wash in distilled water was done. A Philips Tecnai 12 BioTwin microscope (Eindhoven, Netherlands) at 80 kV was used to examine the sections stained with aqueous 1% uranyl acetate for 5 min.

### Nanoparticle Tracking Analysis

A more precise size determination of the EV was obtained through performing NTA, which is a higher resolution technique than electron microscopy (Malloy, 2011). It is based on light scattering and Brownian motion behavior, which allows assessing nanoparticle size distribution and abundance of samples in liquid suspensions (NanoSight NS300, Malvern). The samples were diluted in sterile PBS allowing that the particle concentration is within the linear dynamic range of the equipment (10^6^–10^9^ particles/ml). The analysis was performed using the 532 nm laser, 565 nm long-pass filter, with a camera level set at 9 and a detection threshold of 3.

### Western Blotting

Western blots were used to analyze the protein composition of P100 pellets. We used primary antibodies: (dilution used for all primary antibodies 1/300) for, GPR177 (MyBioSource, Catalog Number: MBS769833; Abcam, GM130 Catalog Number: ab52649; Thermo Fisher Scientific Inc., Wnt3a Catalog Number: PA5-44946; Abcam, Wnt5a Catalog Number: ab174963; Thermo Fisher Scientific Inc., Wnt7a Catalog Number: PA5-80231. All the following antibodies were from Santa Cruz Biotechnology, Inc., Wnt5a, Catalog Number: sc-365370; Alix, Catalog Number: sc-53540; TSG101, Catalog Number: sc-7694; NCAM-L1 Catalog Number: sc-374046; Flotillin-1, Catalog Number: sc-133153; Tubulin, Catalog Number: sc-8035; CD63, Catalog Number: sc-5275. Lysate of HT-22 cells were homogenized in RIPA buffer (10 mM Tris–HCl, 1 mM EDTA, 1% Triton X-100, 0.1% sodium deoxycholate, 0.1% SDS, 140 mM NaCl) supplemented with 1 mM PMSF, 7 μg/mL Pepstatin, 5–10 μg/mL Leupeptin, and 10 μg/mL Aprotinin. The protein content of the cell lysate and the P100 were determined by Pierce^TM^ BCA Protein Assay Kit. An aliquot containing 30 μg of protein in both the lysate and the P100 was mixed with standard sample loading buffer loaded and ran in a 10% acrylamide gel. Proteins were transferred on to a PVDF membrane overnight at 4°C.

### Wnt-C59 Treatment

HT-22 cells were cultured as described before to obtain the conditioned medium, but in parallel, the cells were incubated with 1 μM of the Wnt-C59 Porcupine inhibitor (Tocris, Catalog Number: 5148). The fresh drug was added after 24 h, and the CM was collected after 48 h to perform the enrichment procedure of EV as indicated above. As a stock of 20 mM of Wnt-C59 was prepared in DMSO, control cells were treated with 0.005% DMSO (vehicle) prepared in indicated cell media.

### Proteinase K Assay

The sEVs were isolated by differential ultracentrifugation and resuspended in PBS. Samples were incubated in either PBS or 1.2 μg/ml Proteinase K (Promega, Catalog Number: V3021) in PBS, with or without 0.5% Triton X-100, in a final volume of 40 μl per sample for 5 min at 4°C. The assay was stopped by the addition of 8 μl 5X SDS-PAGE Loading Buffer and heating for 8 min at 97°C, and samples were analyzed by SDS-PAGE and immunoblotted.

### Statistics

The GraphPad Prism program was used for statistical analysis. Data derived from the western blot analysis of the Wnt ligands and other proteins were subjected to the Shapiro–Wilk test, which tested the normality of a data set since the sample size was less than 50. After confirming that all the data were normally distributed, a mismatched *t*-student test was performed. Error bars show SD. No sample size calculation was performed. No exclusion criteria were pre-determined.

## Results

### Characterization of Extracellular Vesicles Released by the Hippocampal HT-22 Cell Line

**FIGURE 1:**
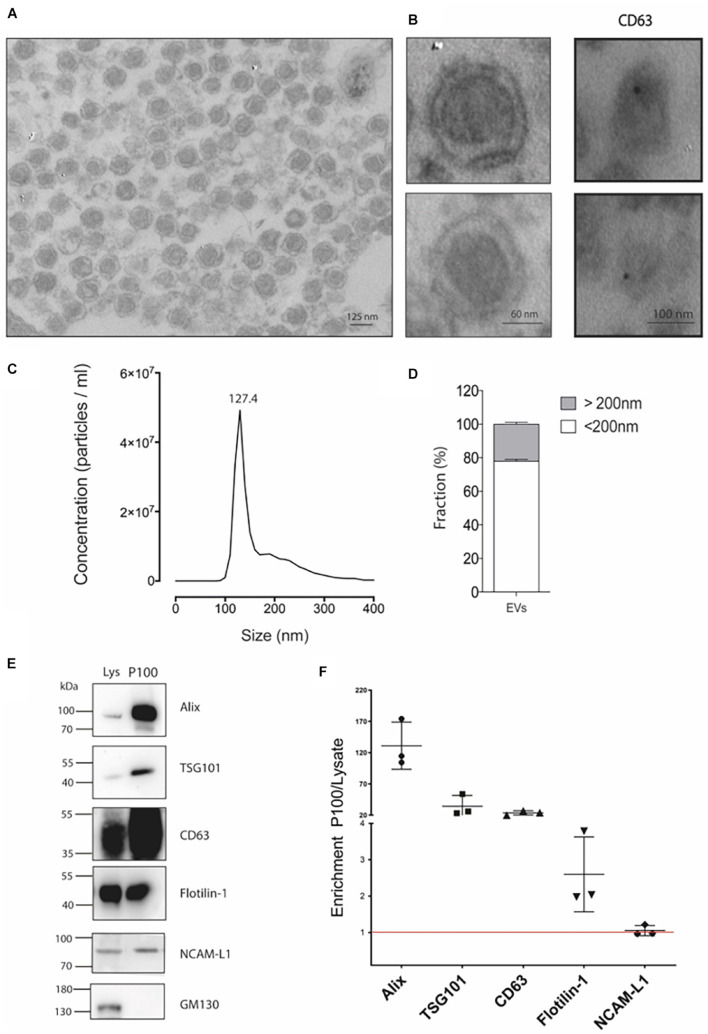
Detailed characterization of P100 vesicles secreted from hippocampal HT-22 cells. **(A)** Representative transmission electron microscopy of P100 shows abundant vesicles with exosome-like appearance with an average diameter of 124.1 ± 6.8 nm of (*n* = 31; three independent cell culture preparations). **(B)** Close capture of single exosomes (left) and immunogold labeling of a selective exosomal biomarker, CD63 (right panel, black dot). **(C)** Nanoparticle tracking analyses show a mean peak at 127.4 ± 1.3 nm (*n* = 5; five independent cell culture preparations). **(D)** Abundance analysis shows that 77.88 ± 2.41% of the vesicles have a diameter of <200 nm and a 22.12 ± 2.41% >200 nm (*n* = 4; four-five independent cell culture preparations). **(E,F)** Western blot and Analysis enrichment (fold) of the protein content of the exosomal markers Alix, 131.08 ± 37.7; TSG101, 34.36 ± 17.00; CD63, 23.82 ± 3.50; Flotillin-1, 2.60 ± 1.03; NCAM-L1, 1.05 ± 0.14. The control GM130, a Golgi-associated protein, was absent. Enrichment is relative to their levels in the lysate. The values were obtained from three independent cell culture preparations.

To isolate extracellular vesicles, we used the mouse hippocampal cell line HT-22, a relevant model for studying glutamate and heavy metal neurotoxicity (Murphy et al., 1989; Fukui et al., 2009). The cell line HT-22 is accessible to culture and provides an *in vitro* model for biochemistry studies requiring large amounts of starting material. In fact, six to eight 150-mm culture plates were required to obtain 100 μg of protein in the P100 pellet. Transmission electron microscopy (TEM) was performed to morphologically characterize the vesicles in the P100 fraction (see section “Materials and Methods”). A representative image of the P100 pellet shows an enriched fraction of exosome-like vesicles containing a membrane with typical bilayer morphology and with an average diameter of 124.1 ± 6.8 nm (Figures 1A,B). Immunogold labeling allows us to visualize the CD63 exosomal signature marker (Figure 1B, right panel). The structure of the vesicles was partially lost due to a mild fixation used in immunogold studies to preserve protein epitopes. Considering that the fixation and dehydration of the sample for TEM analysis can cause deformation of the vesicles, a more precise size determination was obtained using NTA. Consistently, the NTA size distribution was similar to the reported size by TEM. The NTA profiles showed a peak of abundant vesicles of 127.4 ± 1.3 nm (Figure 1C; mode) and a population of vesicles with a size of <200 nm (defined as small extracellular vesicles, sEVs) representing 78% of the particles in the sample. The remaining 22% corresponds to medium or large extracellular vesicles (Figures 1C,D).

Then, we characterize the vesicles released by the HT-22 cells according to the International Society for Extracellular Vesicles (ISEV; Théry et al., 2018). Figure 1E shows a western blot analysis of the P100 protein content profile. The P100 pellet exosomal marker content was enriched relative to that of the lysate in the following rank order: Alix > TSG101 > CD63 > Flotillin-1 (Figure 1F). The neuronal origin of the vesicles released by HT-22 cells is corroborated by the presence of the adhesion molecule NCAM-L1, an accepted exosome biomarker of neuronal origin (Fauré et al., 2006; Figure 1F). Nevertheless, the *cis-*Golgi matrix protein, GM130, was absent from sEVs (Figure 1E), suggesting our preparation was not contaminated with intracellular membranes. Thus, our analysis revealed that vesicles released by HT-22 cells and collected in the P100 fraction effectively correspond to small extracellular vesicles like exosomes (size <200 nm) (Théry et al., 2018).

### Canonical and Non-canonical Wnt Ligands Are Present in Small Extracellular Vesicles

**FIGURE 2:**
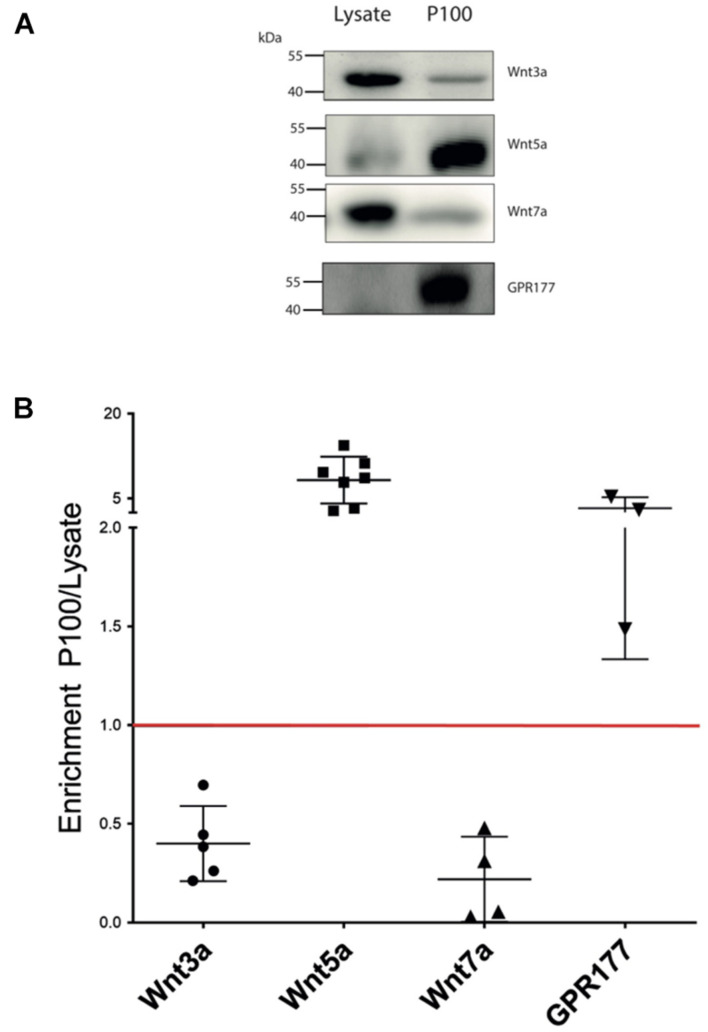
Wnt ligand expression levels in HT-22 cells. **(A)** Comparison of western blot analysis of Wnt3a, Wnt5a, Wnt7a, and GPR177 protein content in cell lysate and P100 fraction. Each lane was loaded with 30 μg protein. **(B)** Summary densitometric measurement of band intensity showing enrichment (fold) of each protein in the P100 pellet relative to a corresponding lysate. Wnt3a, 0.4 ± 0.19 (*n* = 5); Wnt5a, 8.70 ± 4.14 (*n* = 7); Wnt7a, 0.22 ± 0.21 (*n* = 4; four independent cell culture preparations); GPR177 3.30 ± 1.93 (*n* = 3; independent cell culture preparations).

**FIGURE 3:**
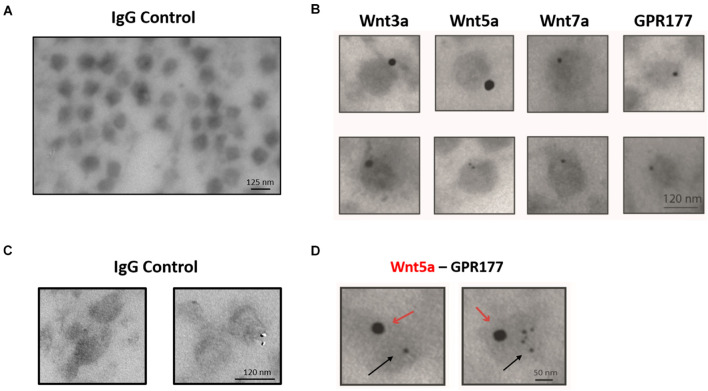
Immunogold-TEM characterization of HT-22 exosomes. **(A)** IgG control for single immunogold labeling. **(B)** Single immunogold labeling shows that exosome-like vesicles secreted by HT-22 cells express Wnt3a, Wnt5a, Wnt7a, and GPR177. **(C)** IgG control for double immunogold. **(D)** Doubled immunogold analysis detected co-expression of Wnt5a and GPR177. Red and black arrows show the presence of Wnt5 and GPR177, respectively.

The enrichment and homogeneous population of exosome-like vesicles prompt us to continue with this cell model to study the presence of Wnt ligands on those vesicles. Next, we examined whether the canonical Wnt3a and Wnt7a ligands and the non-canonical Wnt5a (Korkut et al., 2009; Beckett et al., 2013) were secreted in association with sEVs released by HT-22 cells. Western blot analysis of the sEVs containing fraction (P100) showed enrichment of Wnt5a and the Wnt chaperone, GPR177/Evi, relative to the cell lysate (Figures 2A,B). On the other hand, although present, Wnt3a and Wnt7a were not enriched in this fraction (Figures 2A,B). To corroborate the presence of Wnts in sEVs, immunogold analysis was performed, revealing that Wnt3a, Wnt5a, Wnt7a, and GPR177/Evi were present in the sEVs (Figure 3B). Also, a double immunogold analysis was performed, revealing that Wnt5a (red arrows) and GPR177/Evi (black arrows) were jointly secreted together by the same sEVs (Figure 3D). On the contrary, the IgG respective controls did not show any electrodense mark (Figures 3A,C). Although these data provide evidence of the presence of Wnt ligands in HT-22 secreted vesicles with exosomes characteristics, it was not possible to detect double labeling of Wnt3a or Wnt7a with GPR177/Evi.

### Treatment of HT-22 Cells With the Porcupine Inhibitor Wnt-C59 Affects Wnt Ligands Content of Small Extracellular Vesicles

**FIGURE 4:**
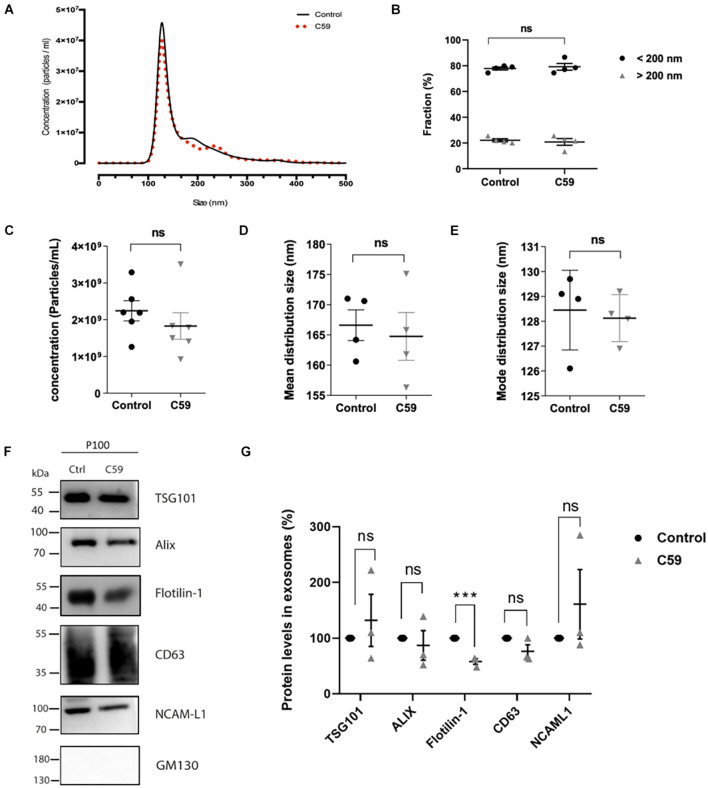
Nanoparticle tracking analyses of exosomes secreted by control and Wnt-C59 treated HT-22 cells and the effect of Wnt-C59 on the content of exosomal markers in the P100 fraction. **(A)** Representative Nanoparticle tracking analyses show a mean peak for both control and Wnt-C59 samples. **(B)** Abundance analysis shows that 80% of the vesicles have a diameter of <200 nm (Control: 77.88 ± 2.41%, Wnt-C59: 79.20 ± 5.22; *n* = 4) and a 20% >200 nm (Control: 22.12 ± 2.41%, Wnt-C59: 20.83 ± 5.22; *n* = 4). **(C)** Concentration of particles. Shapiro–Wilk and KS tests showed that the data conformed to a normal distribution. Subsequently, the result of the Student unpaired *t*-test was *p* = 0.8272 with values of 2.3 × 10^9^ ± 7.6 × 10^8^ (control; *n* = 7) and 1.9 × 10^9^ ± 8.3 × 10^8^ (Wnt-C59; *n* = 7). **(D)** Mean distribution size of the total extracellular vesicles. Shapiro–Wilk and KS tests showed that the data conformed to a normal distribution. Subsequently, the result of the Student unpaired *t*-test was *p* = 0.7602 with values of 166.6 ± 2.5 (control; *n* = 7) and 164.8 ± 3.9 nm (Wnt-C59; *n* = 7). **(E)** Mode distribution size. As in **(D)**, values conform with a normal distribution. The student unpaired *t*-test result was *p* = 0.7824 with values of 128.5 ± 0.8 nm (control; *n* = 7) and 128.1 ± 0.4 nm (Wnt-C59; *n* = 7). *n* value means the number of independent cell culture preparations. **(F)** Representative western blot analysis of the protein content of TSG101, Alix, Flotillin-1, CD63, NCAM-L1 in P100 from control and C59 treated HT-22 cells. GM130 purity control is absent in the P100 fraction. **(G)** Relative protein expression levels of exosomal markers. The staining intensity of each band derived from control P100 was assigned a value of 100%, and the value obtained in the P100 from Wnt-C59 treatment was compared with its respective control. Flotillin-1 was the only exosomal marker whose expression level underwent a significant decrease in exosomes secreted by WntC59 treated HT-22 cells. TSG101 (Control: 100%, Wnt-C59: 132 ± 81.3%; *n* = 3; *p* = 0.700); Alix (Control: 100%, Wnt-C59: 87 ± 45.9%; *n* = 3; *p* = 0.6496); Flotillin-1 (Control: 100%, Wnt-C59: 58 ± 8.7%; *n* = 3; *p* = 0.0011); CD63 (100%, Wnt-C59: 76.3 ± 20.6; *n* = 3; *p* = 0.0952); NCAM-L1 (Control: 100%, Wnt-C59: 161 ± 157.9; *n* = 3; *p* = 0.3831). Mann–Whitney non-parametric *t*-student test (ns, not significant; ***p < 0.005).

Porcupine O-acyltransferase is an O-acyltransferase whose activity affects Wnt ligand secretion in Wnt-producing cells (Kadowaki et al., 1996; Torres et al., 2019). It is unknown if inhibition of PORCN by Wnt-C59 affects only a single or all the secretory pathways that mediate Wnt release from cells (Proffitt et al., 2013; Bengoa-Vergniory et al., 2014). In the present work, we specifically assessed the effect of Wnt-C59 on Wnt ligand secretion associated with sEVs. Although this inhibitor should not affect the exosome formation and secretion pathways, we carried out control experiments. From now on, we will call control and Wnt-C59 the vesicles derived from the vehicle (DMSO) and Wnt-C59 treated cells, respectively. First, we evaluated whether treatment of HT-22 cells with Wnt-C59 alters the relative abundance of each of the vesicle subpopulations released by these cells. NTA was performed with the NanoSight NS300 system to analyze the microvesicle fraction content in the P100 pellet. Figure 4A provides a graphical description of the relationship between particle density (i.e., particles/ml) and their size distribution (nm). There were no significant differences between the control and Wnt-C59 extracellular vesicles (Figure 4B). For both the control and Wnt-C59, there is an enrichment of vesicles with a size of less than 200 nm, similar to the finding showed in Figure 1C. Even though there was a trend toward a decrease in the density of total particles, this decline was not significant (Figure 4C). Similarly, no significant differences were observed in the mean distribution size (Figure 4D) and the mode distribution size (Figure 4E) between the control and Wnt-C59 vesicles. The mean distribution sizes were 166.6 ± 2.5 nm and 164.8 ± 3.9 nm for the control and Wnt-C59 vesicles, respectively (Figure 4D). Their mode distribution size was 128.5 ± 0.8 nm and 128.1 ± 0.4 nm for the control and Wnt-C59 vesicles, respectively (Figure 4E). Therefore, Wnt-C59 did not affect the secretion of sEVs by HT-22 cells.

Western blot analysis was then used as a second control experiment to determine whether Wnt-C59 alters the content of exosomal markers in the sEVs (Figure 4F). As concentration standards have not been described for sEVs protein normalization (Koritzinsky et al., 2017), a statistical analysis of the sEVs protein content with three replicates were performed as shown by others (Imjeti et al., 2017) in which the staining intensity of each band derived from non-treated samples was assigned a value of 100%. The value obtained in the Wnt-C59 sEVs, derived for treated cells, was compared with its respective non-treated control. A *t*-student test was performed on each data pair of a protein. The results show that Alix, CD63, TSG101, and NCAM-L1 expression levels were unaffected by Wnt-C59 treatment (Figures 4F,G). On the other hand, Wnt-C59 treatment significantly decreased (*p* = 0.0011) the Flotillin-1 exosomal content relative to its content in the Wnt-C59 exosomes. The purity of the sEVs preparation was corroborated by the absence of the Golgi protein GM130. Hence, the porcupine inhibitor Wnt-C59 has no effect on the amount, size distribution, or content of exosomal markers but Flotillin-1 in the sEVs.

**FIGURE 5:**
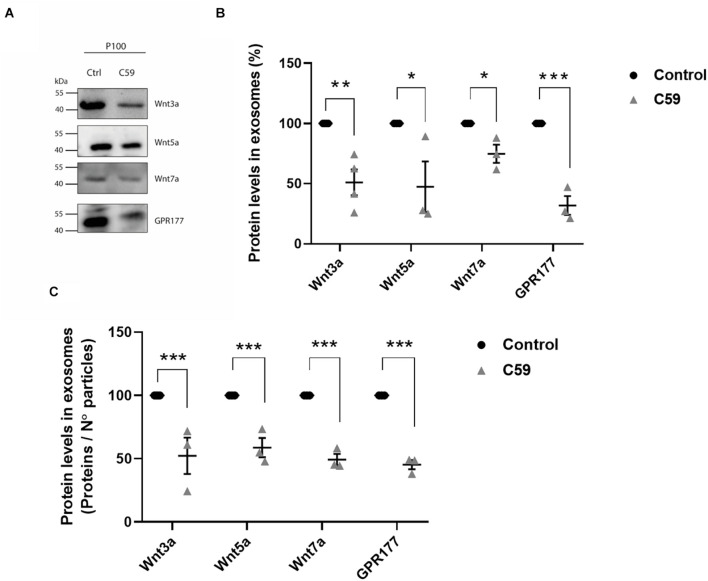
Effects of Wnt-C59 on the content of Wnt ligands in the P100 fraction. **(A)** Comparison of the western blot analysis of Wnt3a, Wnt5a, Wnt7a, and GPR177 protein expression levels in the P100 fraction derived from control and Wnt-C59 treated cells (each lane was loaded with 30 μg protein). **(B)** Relative protein expression levels of Wnt ligands and GPR177 in the P100 fraction. The staining intensity of each band derived from control P100 was assigned a value of 100%, and the value obtained in the P100 from Wnt-C59 treatment was compared with its respective control. Wnt3a (Control: 100%, Wnt-C59: 51.1 ± 21.5%; *n* = 3; *p* = 0.0039); Wnt5a (Control: 100%, Wnt-C59: 39.1 ± 45.6%; *n* = 3; *p* = 0.0404); Wnt7a (Control: 100%, Wnt-C59: 74.82 ± 13.1%; *n* = 3; *p* = 0.0291); GPR177 (Control: 100%, Wnt-C59: 31.9 ± 13.6%; *n* = 3; *p* = 0.001). **(C)** Protein expression levels of Wnt ligands and GPR177 relative to the number of particles loaded per well. Control P100 was assigned a value of 100%, and the value obtained in the P100 from Wnt-C59 treatment was compared with its respective control. In both types of analysis of **(B,C)**, the Wnt3a, Wnt5a, Wnt7a, and GPR177 contents significantly decreased in the exosomes secreted by Wnt-C59 treated cells relative to their levels in the control cells based on the results of Mann–Whitney non-parametric *t*-student test (ns, not significant; *p < 0.05; **p < 0.01; ***p < 0.005). Wnt3a (Control: 100%, Wnt-C59: 52.4 ± 24.9%; *n* = 3; *p* = 0.0295); Wnt5a (Control: 100%, Wnt-C59: 58.8 ± 13.2%; *n* = 3; *p* = 0.0056); Wnt7a (Control: 100%, Wnt-C59: 49.4 ± 7.8%; *n* = 3; *p* = 0.0004); GPR177 (Control: 100%, Wnt-C59: 45.3 ± 6.2%; *n* = 3; *p* = 0.0001). *n* value means the number independent cell culture preparations.

Next, we performed a western blot analysis to determine if Wnt-C59 altered the content of Wnt3a, Wnt5a, Wnt7a, and GPR177/Evi proteins in the P100 fraction obtained from control cells and Wnt-C59 treated cells (Figure 5A). Quantification was carried out in the same way as it was done for Figure 4. The results showed that Wnt ligands and GPR177/Evi levels decreased significantly (Figure 5B). Then, to confirm this finding, a second analysis was performed where the intensity values of the band were normalized dividing them by the number of particles contained in 30 μg of protein loaded per well, as previously reported by others (Dismuke et al., 2016; Shu et al., 2020). Accordingly, the Student *t*-test results show that the Wnt3a, Wnt5a, Wnt7a, and GPR177/Evi protein content decreased significantly (∼50%) compared to the control group (Figure 5C). Therefore, these declines suggest that palmitoylation is a necessary post-translational modification that allows Wnt ligand secretion by sEVs.

### Localization of Wnt Ligands in Small Extracellular Vesicles

**FIGURE 6:**
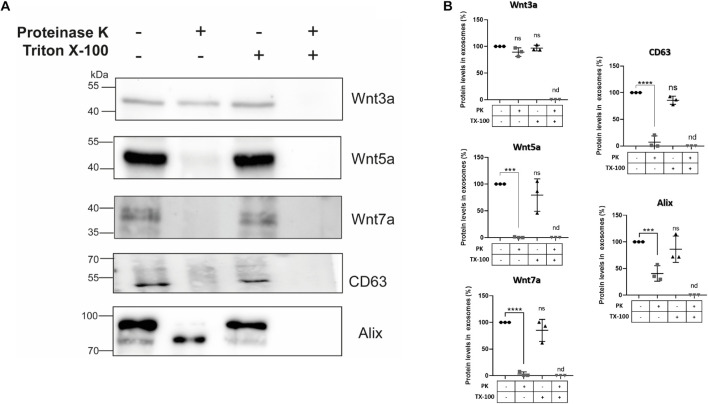
Effects of proteinase K treatment on sEVs Wnt ligand contents. HT-22 cells-derived sEVs were treated with proteinase K in the absence and presence of Triton X-100 (see section “Materials and Methods”). **(A)** Proteinase K treatment did not affect the Wnt3a ligand presence. However, plasma membrane permeabilization with 0.5% Triton X-100 and co-treatment with proteinase K resulted in its disappearance. On the other hand, proteinase K treatment alone resulted in marked declines or even disappearance of Wnt5a, Wnt7a ligands, and the exosomal marker CD63. However, in the case of Alix, a band shift was produced (see comments in the section “Discussion”). Triton X-100 and co-treatment with proteinase K also resulted in the disappearance of CD63 and Alix. **(B)** The graphs show the relative protein expression levels with the different treatments. The staining intensity of each band derived from control P100 was assigned a value of 100%, and the value obtained in the P100 from the different treatments was compared with its respective control (100%). Wnt3a: 89.33 ± 8.02 (PK; *p* = 0.0638); 96.87 ± 5.32 (TX-100; *p* = 0.7695). Wnt5a: 0.41 ± 0.20 (PK; *p* = 0.002); 79.34 ± 30.33 (TX-100; *p* = 0.2904). Wnt7a: 3.02 ± 2.02 (PK; *p* < 0.0001); 85.14 ± 20.65 (TX-100; *p* = 0.2673). CD63: 7.23 ± 7.0 (PK; *p* < 0.0001); 85.73 ± 7.63 (TX-100; *p* = 0.0842). Alix: 40.72 ± 14.59 (PK; *p* < 0.0025); 86.23 ± 24.73 (TX-100; *p* = 0.5365). n.d., not detected; PK, proteinase K; TX-100, triton X-100. Mann–Whitney non-parametric *t*-student test (ns, not significant; ***p < 0.005; ****p < 0.0001). The images are representative of three independent cell culture preparations.

To establish whether Wnt ligands are localized within different regions of sEVs, we used proteinase K to perform a “shaving” procedure (Escrevente et al., 2011; Wang et al., 2017). In this procedure, proteinase K only degrades proteins on the surface of the vesicles, since it does not penetrate the membranous lipid bilayer. However, if proteinase K and Triton X-100 are applied together, the detergent lyses the outer limiting vesicles membrane rendering all sEVs proteins accessible to proteolysis by proteinase K. Hence, the effect of proteinase K on sEVs protein content in the presence and absence of Triton X-100 was compared (see section “Materials and Methods”). Western blot data shows that proteinase K treatment alone did not degrade Wnt3a but Wnt5a, and Wnt7a were degraded (Figure 6). On the other hand, co-incubation with proteinase K plus Triton X-100 completely digested all these proteinaceous Wnt ligands. As a control we used the exosomal biomarker CD63 which is a tetraspanin transmembrane protein (Théry et al., 2018) sensitive to proteinase K treatment as demonstrated by others (Vlassov et al., 2012; Phoonsawat et al., 2014). As luminal control we used Alix which was still present after proteinase K digestion but underwent a shift to a lower molecular weight protein. A similar result was found for Alix for exosomes derived from neurons treated with proteinase K (Wang et al., 2017). Moreover, the presence of two bands for Alix has been previously documented in several cell lines (Shtanko et al., 2011; Bongiovanni et al., 2012; Lopes-Rodrigues et al., 2019), with the 97 kDa band representing the full-length protein and the 75 kDa band would represent a cathepsin digestion product (Impens et al., 2010; Lopes-Rodrigues et al., 2019) that have been found in exosomes (Fiandaca et al., 2015; Kanninen et al., 2016).

Taken together, these results are unique since they suggest that Wnt ligands are segregated into different compartments of exosome-like vesicles secreted by HT-22 cells. Accordingly, it is likely that Wnt3a is restricted to the luminal interior, while Wnt5a and Wnt7a are delimited to the outer limiting membrane.

## Discussion

NTA analysis of the P100 pellet vesicular fraction showed that approximately 80% of all the particles have an average diameter of 127.4 nm, which is within the range described for exosomes (Raposo and Stoorvogel, 2013). Furthermore, electron microscopic analysis showed a highly enriched type of vesicle (average diameter 124.1 nm) with a well-defined round shape similar to exosomes (Figure 1). As there are no specific and universal signature biomarkers of exosomes needed to confirm their presence, we instead relied on a battery of markers that have been suggested to be indicative of small extracellular vesicles (Théry et al., 2018). In this study, we used several sEVs markers proposed by Théry et al. (2018) in two categories, those belonging to category 1 that are indicative of lipid bilayer structure sandwiched between the outer membrane transmembrane proteins in the sEVs, like CD63, revealing the presence of tetraspanins. Furthermore, category 2 markers such as TSG101, Flotillin, and Alix reflect the cytosolic proteins involved in sEVs biogenesis. As the evaluation of the marker expression profile assesses the purity of preparation, we also quantified: (a) the GM130 expression level which is a *cis-*Golgi matrix protein (Théry et al., 2018); (b) tubulin the cytoskeletal protein, which were both absent from our micro-vesicular preparation indicating intracellular contaminants were entirely discarded following cell rupture (not shown). The confirmed presence of extracellular vesicles coupled with the exosome-like particle diameter morphology and biomarker content allows us to conclude that the particles are exosomes. Also, our purified sEVs showed expression of NCAM-L1, a neuronal adhesion molecule critical for neuronal development and found in exosomes released from neurons (Fauré et al., 2006). Concerning the Wnt3a, Wnt5a, and Wnt7a ligands, all were associated with the P100 fraction. However, only Wnt5a and GPR177/Evi were enriched, which is consistent with immunogold-TEM (Figure 3).

Wnt ligands are highly hydrophobic molecules that undergo acylation during their intracellular trafficking by the action of the enzyme PORCN (Torres et al., 2019). A role for PORCN in Wnt signaling was first suggested based on the similarity between the phenotype generated by Wnt gene mutations and that caused by a mutation in the PORCN gene of *Drosophila* (van den Heuvel et al., 1993; Kadowaki et al., 1996). Palmitoylation of Wnt ligands by the O-acyltransferase PORCN is considered essential for Wnt secretion (Barrott et al., 2011; Biechele et al., 2011), but this paradigm was challenged recently (Richards et al., 2014; Rao et al., 2019). Rao et al. (2019) showed that inhibition of the PORCN sometimes does not phenocopy the loss of specific Wnt molecules in some cancer cells, and both Wnt3a as Wnt4a secretion was PORCN independent. A PORCN-independent Wnt secretion and signaling was also observed in CD8+ T cells (Richards et al., 2014). The latter observation leads to a more specific question, is acylation a requirement for Wnt ligands to be secreted in any of the mechanisms so far documented. Here, we focused on one of those mechanisms describing how Wnt palmitoylation inhibition specifically alters Wnt proteins association with small extracellular vesicles.

First, we exhaustively controlled any side effects of Wnt-C59 on the vesicle subpopulation obtained in the preparations (Figure 4). NanoSight analysis showed that the average diameter of the EVs secreted by control cells and treated with the drug was maintained. Under both conditions, approximately 80% of the vesicles present correspond to sEVs (small extracellular vesicles) whose diameter was less than 200 nm. Also, the concentration of secreted vesicles was not affected. Hence, Wnt-C59 did not affect the sEVs. Similarly, none of the exosome markers used to characterize these vesicles were altered in the presence of Wnt-C59. Surprisingly, we found that the amount of Flotillin-1 associated with sEVs decreases with the treatment of cells with Wnt-C59. Flotillins are hydrophobic proteins located in the inner part of the plasma membrane, where they play a fundamental role in the formation of lipid rafts (Bickel et al., 1997; Rajendran et al., 2007). Also, Flotillin microdomains have been described in recycling endosomes, as well as in exosomes (Meister and Tikkanen, 2014). Interestingly, palmitoylation is necessary for Wnt ligands to associate with lipid rafts (Zhai et al., 2004). Flotillin-2, although not specific for Wnt, is known to participate in the intracellular traffic of Wnt (Katanaev et al., 2008; Solis et al., 2013; Galli et al., 2016). Our observation suggests that there is probably a closer relationship between these proteins with the exocytosis of Wnt ligands. The intracellular trafficking and exocytosis model of Wnt ligands is incomplete, and the role played by proteins as Flotillins must be revisited. Regarding the ligand content, Wnt3a, Wnt5a, and Wnt7 ligands underwent a similar partial decrease in the sEVs derived from HT-22 cells treated with Wnt-C59. The levels of Wnt transport chaperone, GPR177/Evi, in sEVs also diminished with the drug treatment. Note that GPR177/Evi is not a PORCN target; therefore, this finding supports an interdependence among Wnt, GPR177/Evi and PORCN as suggested before (Bartscherer et al., 2006; Korkut et al., 2009; Glaeser et al., 2018). Regarding the ligand content, Wnt3a, Wnt5a, and Wnt7 ligands underwent a similar partial decrease in the sEVs derived from HT-22 cells treated with Wnt-C59. The levels of Wnt transport chaperone, GPR177/Evi, in sEVs also diminished with the drug treatment. Note that GPR177/Evi is not a PORCN target; therefore, this finding supports an interdependence among Wnt, GPR177/Evi, and PORCN as suggested before (Bartscherer et al., 2006; Korkut et al., 2009; Glaeser et al., 2018).

The way Wnt molecules diffuse at the extracellular space is still a subject of debate. One of the mechanisms is via exosomes, but how these vesicles deliver Wnt to a target cell is still unclear. A few studies suggest Wnt association with the outer face of exosomes (Gross et al., 2012; McBride et al., 2017), which mechanistically makes sense considering that Wnt ligands would activate extracellular receptors. Accordingly, we found Wnt5a and Wnt7a associated with the extracellular surface of HT-22 derived sEVs. However, an unexpected finding of the present work was the presence of Wnt3a inside the exosomes. Exosomes contain receptors and ligands at their outer membrane surface, whereas proteins and RNA are encapsulated inside (Théry et al., 2002; Valadi et al., 2007; Doyle and Wang, 2019). A mechanism of brain dissemination for Tau, a protein transported inside exosomes, has recently been postulated. The mechanism suggests that exosomes that contain Tau inside would be released first by a “parent” neuron, then captured intact by the soma of a second neuron through a transcytosis mechanism, which in turn would transport the intact vesicle intracellularly to be released by the axonal terminal. Then, a third synaptically connected neuron would take the exosome by endocytosis, releasing the content in its cytoplasm (Wang et al., 2017). This transcytosis mechanism has also been observed in some cancers, e.g., exosomes would support brain metastasis through transcytosis across the blood-brain barrier (Peinado et al., 2017; Morad et al., 2019). Similarly, under a physiological condition, transcytosis mediated by exosomes accounts for how folate is transported across the blood-cerebrospinal fluid barrier (Grapp et al., 2013). Transcytosis also was observed in vertebrate cells for Wnt3a and Hedgehog, another lipid-modified signaling protein (Gallet et al., 2008; Callejo et al., 2011; Yamamoto et al., 2013). However, none of those studies described the extracellular mechanism transporting the endocytosed/transcytosed Wnt ligand. Therefore, the presence of Wnt3a inside exosomes opens new perspectives to explain long-range actions of Wnt.

## Conclusion

The present work provides evidence that acylation is needed for Wnt ligands association to small extracellular vesicles, that according to our findings, they are exosomes. In addition, we found that Wnt ligands can be associated both to the outer surface and the luminal side of exosomes, which might provide clues to explain the diverse mechanisms of actions postulated for Wnt molecules. Many questions remain about how Wnt ligands induce target activation and signaling transduction. The same uncertainties apply to explain how Wnts are packed into exosomes prior to secretion. Clarifying these questions also requires considering the cellular context and the physiological state of the releasing and receiving cells.

## Data Availability Statement

The original contributions presented in the study are included in the article/supplementary material, further inquiries can be directed to the corresponding authors.

## Author Contributions

VT: conceptualization, methodology, writing-original draft preparation, investigation, supervision, writing-reviewing and editing, and funding acquisition. DB: methodology, writing-original draft preparation, and investigation. MV-G: methodology, investigation, and writing-reviewing and editing. DA: investigation and writing-reviewing and editing. NI: conceptualization, supervision, reviewing and editing, and funding acquisition. All authors contributed to the article and approved the submitted version.

## Conflict of Interest

The authors declare that the research was conducted in the absence of any commercial or financial relationships that could be construed as a potential conflict of interest.

## Publisher’s Note

All claims expressed in this article are solely those of the authors and do not necessarily represent those of their affiliated organizations, or those of the publisher, the editors and the reviewers. Any product that may be evaluated in this article, or claim that may be made by its manufacturer, is not guaranteed or endorsed by the publisher.
